# Accuracy of flow-void diameters on MR images in diagnosing uterine arteriovenous malformations in patients with pregnancy-related diseases

**DOI:** 10.1038/s41598-021-99209-9

**Published:** 2021-10-06

**Authors:** Hui-zhu Chen, Fu-min Zhao, Ling-jun Liu, Xiao-hui Dai, Xue-sheng Li, Gang Ning, Ying-kun Guo

**Affiliations:** 1 Department of Radiology, Key Laboratory of Obstetric & Gynecologic and Pediatric Diseases and Birth Defects of Ministry of Education, West China Second University Hospital, Sichuan University, 20# Section 3 South Renmin Road, Chengdu, 610041 Sichuan China; 2 Department of Ultrasound, Key Laboratory of Obstetric & Gynecologic and Pediatric Diseases and Birth Defects of Ministry of Education, West China Second University Hospital, Sichuan University, 20# Section 3 South Renmin Road, Chengdu, 610041 Sichuan China

**Keywords:** Reproductive disorders, Urogenital reproductive disorders, Techniques and instrumentation, Imaging techniques, Ureter, Diagnosis, Signs and symptoms, Comorbidities, Risk factors

## Abstract

To evaluate the “flow void” diameter in patients with pregnancy-related diseases with and without uterine AVMs and assess the diagnostic performance of unenhanced MRI for uterine AVMs. From May 2014 to April 2019, 79 patients with pregnancy-related diseases were included, including 36 with and 43 without uterine AVMs confirmed by DSA. On MRI, the diameter of the most prominent “flow void” (hereinafter referred to as fv-D) was measured and compared between patients with and without uterine AVMs. The diagnostic performance of fv-D was estimated with receiver operating characteristic curves. The “flow void” sign was observed in patients with and without uterine AVMs (P > 0.05). The fv-D was significantly larger in patients with uterine AVMs in the myometrium and parametrium than in patients without uterine AVMs (P < 0.0001). The fv-D achieved a reliable diagnostic performance in the myometrium (sensitivity 80.6%, specificity 60.5%, negative predictive value 78.8%, positive predictive value 63%, AUC 0.727, cut-off: > 1.33 mm) and parametrium (sensitivity 97.2%, specificity 67.4%, negative predictive value 96.7%, positive predictive value 71.4%, AUC 0.881, cut-off > 2.6 mm). On MRI, fv-D could diagnose uterine AVMs. The fv-D had a much higher diagnostic efficiency in the parametrium than in the myometrium. The parametrium fv-D greatly improved the diagnostic sensitivity and provides a more accurate, noninvasive method of investigating possible uterine AVMs.

## Introduction

Uterine arteriovenous malformation (AVM) is a rare cause of heavy and sometimes life-threatening vaginal bleeding and is characterized by an abnormal connection between arterial and venous circulation. The incidence of uterine AVMs is unknown^1,2^, but recent investigators have speculated that the true incidence of uterine AVMs may be much higher than that reported in the literature because of the increase in intrauterine interventions (those following pregnancy, caesarean section, curettage and abortion), the availability of colour and spectral flow Doppler and the higher index of suspicion; hence, uterine AVMs might not be as rare as previously thought^3,4^. AVMs are classified as either congenital or acquired. Congenital AVM results from a failure in embryological vascular differentiation^5–9^. However, most cases of uterine AVMs are acquired and may occasionally coexist with pregnancy-related conditions, such as retained products of conception (RPOC)^3,10–12^, gestational trophoblastic disease (GTD)^13^ and caesarean scar pregnancy (CSP)^14^. RPOC initially consists of retained villi, and then the residual villi undergo necrosis with fibrin deposition, producing a pathological condition called placental polyps; these polyps are accompanied by frequent and abundant vascularization in the myometrium attached to the remnant tissue, thus leading to the formation of arteriovenous fistulas and subsequent development of an arteriovenous communication^15^. CSP is the most common aetiology for acquired uterine AVMs^16,17^. Kim et al. proposed that uterine AVMs may result from the erosive nature of syncytiotrophoblastic tissue and chorionic villi during placentogenesis in patients with CSP, during which a faulty decidual layer induces the generation of abnormal connections between the arterial and venous circulation^18^. In patients treated for CSP, the gestation that is left in place could be considered an RPOC and can present a risk for the development of an AVM^14^. In patients with GTD, uterine AVMs can develop as a result of destruction of the uterine vasculature by trophoblastic invasion of the spiral arteries^19^. Dilatation and curettage (D&C) is method for evacuating RPOC, noninvasive hydatidiform moles and CSP; however, if these conditions coexist with uterine AVMs, massive, life-threatening bleeding can occur, and D&C is therefore contraindicated in these cases^8,19,20^. An accurate assessment of pregnancy-related diseases coexisting with uterine AVMs is critically important for selecting a treatment protocol and the optimal timing of treatment.

Digital subtraction angiography (DSA) is regarded as the gold standard for the diagnosis of uterine AVMs^21^. However, DSA is rarely used for diagnostic purposes alone because of the associated radiation and use of iodinated contrast agents^22^. More recently, transvaginal ultrasound (TVUS) has been traditionally considered the imaging modality of choice for the initial assessment and characterization of uterine AVMs. Colour Doppler imaging can identify and localize areas of increased vascularity, whereas spectral flow Doppler generates a waveform from which systolic and diastolic velocities can be measured and shows low-resistance, high-velocity flow^23–25^. However, TVUS is less quantitative, the image quality is operator-dependent, and the reduced field of view and blind areas caused by air in the bowel sometimes occur. Furthermore, in complex cases and adjacent organ involvement, the limited field of view on ultrasound may hamper accurate characterization of disease extent.

Magnetic resonance imaging (MRI) plays an important role in evaluating the extent of lesions, particularly deeper lesions, and their relationship to adjacent structures^26^. Furthermore, on MRI, flow voids, which result from signal loss due to high velocity, turbulence and odd-echo dephasing, are highly correlated with the presence of pathological vessels^27^. Additionally, arteries, aneurysms, and AVMs can be identified on MRI without contrast material^27,28^. In clinical practice, we found that patients with uterine AVMs had a much larger degree of dilatation of the flow voids in the uterus or parametrium than patients without uterine AVMs. Therefore, we hypothesized that the diameter of the flow void may improve the diagnostic accuracy of uterine AVMs.

Nevertheless, few articles have evaluated the use of MRI for identifying uterine AVMs or the coexistence of uterine AVMs and pregnancy-related diseases, and the available studies have been limited to isolated case reports or small case series^6,10,29–32^. Therefore, the purpose of our study was to evaluate the diagnostic performance of myometrial and parametrial flow void diameter measurements on MRI for the diagnosis of AVMs using DSA as a reference standard.

## Results

### Patient demographics and the morphological characteristics of pregnancy-related diseases on MRI

A total of 79 selected women aged 19–44 years (mean age 31 years; SD 6.19) were enrolled. The clinical presentations were vaginal bleeding, abdominal pain and/or masses in the uterine cavity and myometrium after abortion or parturition. Serum β-human chorionic gonadotropin (HCG) values were 0–191,103.3 mIU/L (median 7042.15 mIU/L) before the MR examinations in all patients. The duration between MRI and DSA ranged from 0.04 to 17 days (2.17 ± 3.58 days). The time between abortion/parturition and MRI study range from 1 to 270 days (27.88 ± 38.66 days). The pertinent characteristics of the patients are summarized in Table 1.

**Table 1:** Patient characteristics.

The number of patient	With uterine AVM (n = 36)	Without uterine AVM (n = 43)	P
Age (year)	22–44 (32.3 ± 5.7)	19–43 (31.4 ± 5.3)	> 0.05
HCG level at diagnosis (mIU/L)	0–191,103.3 (median 3639.9)	0–138,162.1 (median 9459.6)	> 0.05
**History of pregnancy**			
Gravidity	1–8 (4.1 ± 1.5)	1–10 (4.1 ± 2.1)	> 0.05
Cesarean delivery	0–3 (1.0 ± 0.7)	0–2 (1.0 ± 0.6)	> 0.05
Abortion	1–6 (3.0 ± 1.3)	0–8 (3.0 ± 2.0)	> 0.05
Inter time between MRI and DSA (day)	0.04–13 (1.2 ± 2.2)	0.08–17 (2.9 ± 4.3)	> 0.05
Inter time between abortion/parturition and MRI (day)	1–270 (34.3 ± 49.3)	1–120 (22.5 ± 26.9)	> 0.05
Bleeding	61.1% (22/36)	58.1% (25/43)	> 0.05
Abdominal pain	13.9% (5/36)	7% (3/43)	> 0.05
**RPOC**	26	29	–
Disease duration (day)	1–93 (30.9 ± 26.2)	1–120 (27.0 ± 29.4)	> 0.05
In uterine cavity	11	15	–
In cesarean scar	15	14	–
**Cesarean scar pregnancy (CSP)**	8	13	–
Gestational age (week)	5.6–11.4 (7.8 ± 1.9)	5.6–12.2 (7.5 ± 1.7)	> 0.05
**Gestational trophoblastic tumor (GTD)**	2	1	–
Disease duration (day)	270, 120 (mean, 195)	36	–

*RPOC* retained products of conception, *GTD* gestational trophoblastic disease, *CSP* caesarean scar pregnancy, *HCG* human chorionic gonadotropin.

With MRI, 54 patients were diagnosed with RPOC, and its typical feature was a bulky uterus with a soft tissue mass in the uterine cavity (26/54) and in the caesarean scar (29/54). There was effacement of the junctional zone, myometrial invasion, haemorrhage and multiple vascular-like irregular, serpentine flow void signs in the myometrium and parametrium. Contrast-enhanced MRI was performed for 22 patients and showed intense uterine enhancement with enhanced RPOC projecting into the endometrial cavity or caesarean scar.

Twenty-two patients were diagnosed with CSP. MRI showed an abnormal cystic component signal in the anterior wall of the uterine isthmus at the incision. The myometrium at the incision scar was either not continuous, with the thinnest area measuring approximately 0.1–0.3 cm, or was absent between the gestational sac (GS) and bladder. The lesions showed low signal on T1WI and mixed signal on T2WI and were prominent in the uterine cavity and multiple vascular-like irregular, serpentine flow void signs in the myometrium and parametrium.

**Figure 1:**
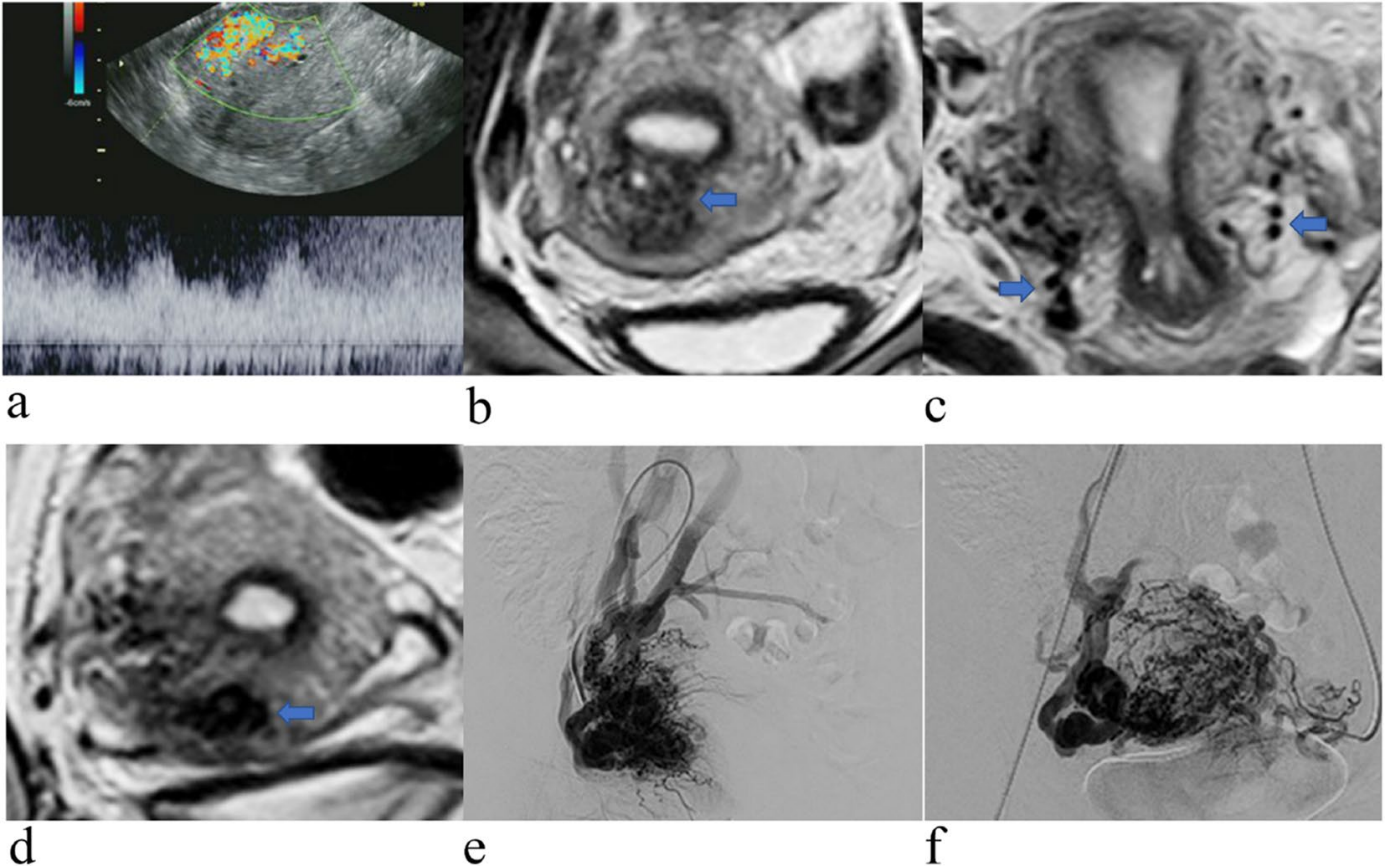
Images obtained of a 29-year-old woman with GTD coexisting with a uterine AVM after chemotherapy. (**a**) Colour Doppler imaging of the arteriovenous malformation within the myometrium showing brisk, highly expanded flow and pulsed Doppler imaging showing low-resistance, high-velocity flow. (**b**) Coronal T2-weighted MRI showing a mass located in the myometrium with an unclear boundary and heterogeneous signal (blue arrow). (**c**) Axial showing multiple vascular-like, irregular, serpentine flow void signs in the parametrium (blue arrow). (**d**) Coronal T2-weighted MRI showing multiple vascular-like, irregular flow void signs in the myometrium (blue arrow). (**e**,**f**) Pelvic angiogram of the left and right uterine arteries revealed simultaneous filling of the right draining veins in the early arterial phase, which is a definitive diagnostic finding of uterine AVMs.

Three patients were diagnosed with GTD. MRI showed that the lesions were located in the myometrium with unclear boundaries, heterogeneous signals and multiple vascular-like irregular serpentine flow void signs in the myometrium and parametrium (Fig. 1).

### The probability of flow void signs on MRI

In our study, flow voids could be observed in the parametrium in all patients with uterine AVMs and in 38/43 patients without uterine AVMs. In the myometrium, flow voids could be observed in 35/36 patients with uterine AVMs and in 39/43 patients without uterine AVMs. The probabilities of a flow void sign appearing in the myometrium (uterine AVM, 97.2% vs. control, 90.7%, P > 0.05) and parametrium (uterine AVM, 100% vs. control, 88.4%, P > 0.05) were determined (Table 2).

**Table 2:** Prevalence and mean diameter of flow voids in women with and without uterine AVMs.

Location	Probability (%)		P value	Diameter (mm)		P value
	With AVM	Without AVM		With AVM	Without AVM	
Myometrium	97.2% (35/36)	90.7% (39/43)	> 0.05	2.66 ± 1.65	1.41 ± 1.27	< 0.0001
Parametrium	100% (36/36)	88.4% (38/43)	> 0.05	5.07 ± 2.00	2.31 ± 1.66	< 0.0001

### Diagnostic accuracy of MRI according to fv-D in the myometrium and parametrium

The fv-D was significantly larger in patients with uterine AVMs than in those without AVMs in the myometrium (uterine AVM, 2.66 ± 1.65 mm vs. control, 1.41 ± 1.27 mm, P < 0.0001) and parametrium (uterine AVM, 5.02 ± 2.00 mm vs. control, 2.31 ± 1.66 mm, P < 0.0001) (Table 2).

**Figure 2:**
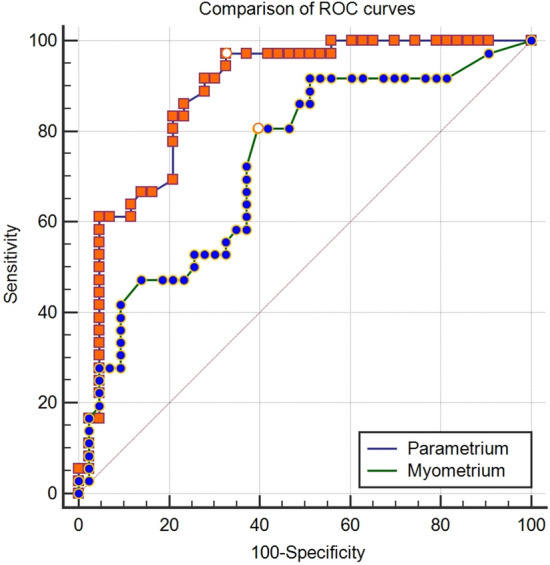
The ROC curves of fv-D values in the parametrium and myometrium.

The fv-D validity values in the myometrium (sensitivity 80.6%, specificity 60.5%, negative predictive value 78.8%, positive predictive value 63%, AUC 0.727, cut-off: > 1.33 mm) and parametrium (sensitivity 97.2%, specificity 67.4%, negative predictive value 96.7%, positive predictive value 71.4%, AUC 0.881, cut-off > 2.6 mm) were calculated (Table 3). The ROC curves are shown in Fig. 2.

**Table 3:** The diagnostic accuracy of fv-D in the myometrium and parametrium.

	Se	Sp	+ PV	− PV	AUC	95% CI	P value	YI	Cut-off (mm)
Myometrium	80.6	60.5	63	78.8	0.727	0.616–0.822	< 0.0001	0.410	> 1.33
Parametrium	97.2	67.4	71.4	96.7	0.881	0.789–0.943	< 0.0001	0.647	> 2.6

AUC = area under the curve, Se = sensitivity, Sp = specificity, + PV = positive predictive value, -PV = negative predictive value, YI = Youden index.

### Characteristics of AVMs on DSA

There were no adverse events from performing DSA. 79 patients underwent embolization, and 36 patients were diagnosed with uterine AVMs. DSA demonstrated that uterine arterial angiography detected one or more draining veins on early arterial-phase images (Fig. 1). The other accompanying signs included obvious thickening and increased tortuosity of the blood supply arteries, the presence of a vascular mass in the lesion, and an abnormal increase in blood vessels with tubular or cystic dilation along with more obvious accumulation in the lesion region when the arterial contrast agent began to subside.

### Agreement for fv-D measurements

The intraobserver and interobserver variability of the fv-D measurements were analysed. The ICC for intraobserver variability was 0.93 (95% confidence interval 0.91–0.95), and the ICC for interobserver variability was 0.87 (95% confidence interval 0.82–0.90).

**Figure 3:**
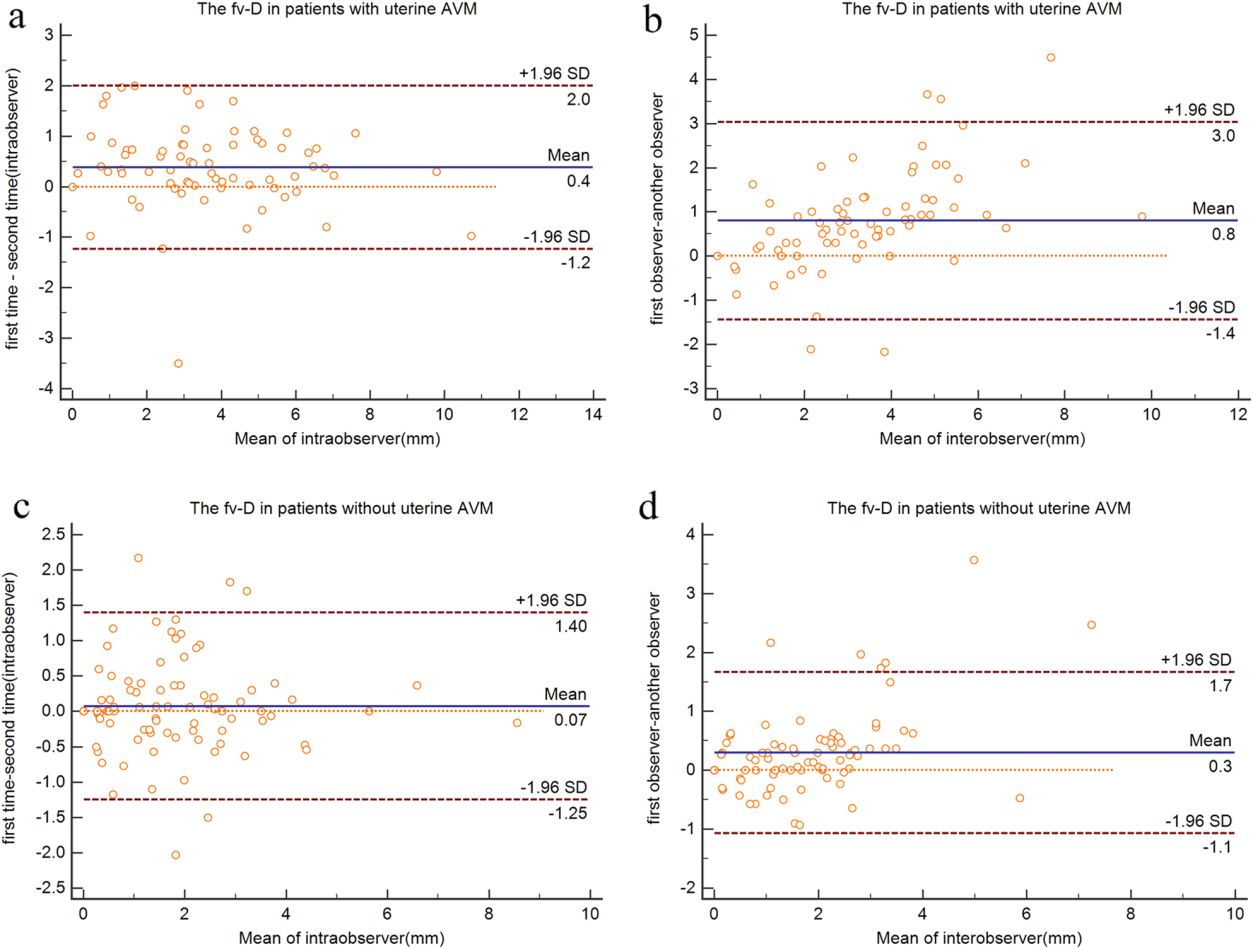
Bland–Altman plot showing agreement in the assessment of fv-D values on MRI. (**a**,**b**) The biases between intra- and interobserver measurements are 0.4 mm and 0.8 mm in patients with uterine AVMs; (**c**,**d**) The biases between intra- and interobserver measurements are 0.07 mm and 0.3 mm, respectively, in patients without uterine AVMs.

Bland–Altman analysis demonstrated good intraobserver and interobserver agreement in patients with uterine AVMs and without uterine AVMs, respectively (Fig. 3).

## Discussion

Our results show that fv-D could be used to diagnose uterine AVMs, and on MRI, the diagnostic efficiency of fv-D in the parametrium (AUC 0.881) is much higher than that in the myometrium (AUC 0.727). The fv-D in the parametrium has an excellent sensitivity (97.22%) and AUC (0.881) for diagnosing uterine AVMs. This information is of clinical importance and could aid in diagnosis and treatment planning processes by indicating a need for preoperative or palliative transcatheter arterial embolization.

As shown in previous reports^3,4,14^, all patients with acquired uterine AVMs were in their reproductive years in our study. Most previously reported acquired uterine AVMs were identified only after ruling out RPOC, GTD or other pregnancy-related diseases^14,33^. However, in contrast to previous reports, our data mainly focused on pregnancy-related diseases coexisting with uterine AVMs, including RPOC (26/36), CSP (8/36) and GTD (2/36). RPOC and CSP are the most common aetiologies for acquired uterine AVMs^15–18^. In patients treated for CSP, gestation that is left in place could be considered an RPOC and can present a risk for the development of AVMs^14^. In our study, among the patients with uterine AVMs, 15 patients with treated CSP were included among the patients with RPOC, and 8 patients with CSP developed AVMs. Hence, in our study, RPOC comprises a large proportion of aetiology for acquired uterine AVMs, especially in patients treated for CSP. At present, TVUS and MRI are often used for identifying uterine AVMs. However, in complex cases and adjacent organ involvement, the limited field of view on ultrasound may hamper accurate characterization of disease extent, especially for pregnancy-related diseases coexisting with uterine AVMs. MRI plays an important role in evaluating the extent of lesions, and the flow void sign is highly correlated with the presence of pathological vessels^27^.

MR angiography (MRA) and dynamic contrast-enhanced MRI are useful in defining the nature of the blood flow within a lesion^3,7,32,34^, but both of them require a long acquisition time, furthermore, dynamic contrast-enhanced MRI needs gadolinium contrast media and it can be useful for both planning therapeutic embolization and monitoring the effects of treatment^8^. In our study, MRI examinations were performed for patients with RPOC, CSP, and GTD, therefore, MRA and dynamic contrast-enhanced MRI could not be available due to the retrospective nature of the study. The flow void sign, such as multiple dotlike, tubular structures or multiple serpentine with low signal intensity on both T1WI and T2WI, could be found in patients with uterine AVMs in the myometrium and parametrium, which is consistent with the findings of several previous studies^15,24,28,32,35,36^. Additionally, in clinical practice, we found that the flow void sign can also be observed in patients without uterine AVMs, and there was no significant difference between patients with and without AVMs (P > 0.05). Nevertheless, most previously reported uterine AVMs on MRI have focused on the morphological characteristics of the flow void sign^15,28,32,36^, and the available studies have been limited to isolated case reports or small case series^6,10,29–32^. In contrast to previous reports, we found that fv-D was often larger in patients with uterine AVMs than in those without uterine AVMs, and this was true for AVMs occurring in both the myometrium and the parametrium (P < 0.001) in our study. Therefore, we quantified the flow void sign by measuring fv-D for diagnosing uterine AVMs in patients with pregnancy-related diseases on MRI. Our results showed that fv-D in the parametrium had a much higher AUC (0.881) for diagnosing uterine AVMs than fv-D in the myometrium (AUC 0.727). An fv-D in the parametrium greater than 2.6 mm could be utilized to diagnose uterine AVMs, and this cut-off had a sensitivity of 97.2% and AUC of 0.881. For fv-D in the myometrium, the diagnostic accuracy is relatively low. This may be associated with large amounts of blood drained through the parametrium vessels, and we speculate that fv-D may be proportional to the shunt volume of uterine AVMs (i.e., a greater volume of shunted blood is associated with a larger diameter). Although the erosive nature of the syncytiotrophoblastic tissue and chorionic villi can increase angiogenesis in the myometrium^15,18^, this phenomenon does not necessarily indicate the formation of an AVM. On the other hand, the dilation of flow voids in the myometrium may be limited by the resistance of the myometrium.

We reported the measurement of fv-D in the myometrium and parametrium on MRI for the first time, and the fv-D value in the parametrium greatly improves the sensitivity of diagnosis and provides a new evaluation index for diagnosing uterine AVMs without the use of contrast medium using T1- and T2-weighted images alone. This measurement could aid gynaecological doctors in planning treatment for patients with pregnancy-related diseases coexisting with uterine AVMs.

Our study has several limitations. First, it included a relatively small number of patients recruited in a single centre. Although the results indicate that fv-D has substantial value in diagnosing uterine AVMs, the reliability of the flow void sign for diagnosing uterine AVMs needs to be further investigated in multiple centres. Second, we were unable to obtain resistance index (RI) calculations and peak systolic velocity (PSV) flow measurements in all patients on TVUS due to the retrospective nature of the study, so we could not compare the diagnostic value of TVUS with that of MRI for uterine AVMs. Finally, selection bias could not be avoided in our study due to its retrospective nature.

In summary, on MRI, the flow void sign can be present not only in patients with pregnancy-related diseases with uterine AVMs but also in those without uterine AVMs. However, a fv-D greater than 2.6 mm (cut-off value) could be used to significantly distinguish uterine AVMs with high sensitivity, and the diagnostic efficiency of fv-D in the parametrium is much higher than that in the myometrium.

## Methods

This single-centre retrospective study was approved by the Institutional Review Board of West China Second University Hospital (No. 2020174), and we pledged to abide by the declaration of Helsinki (2013 EDITION) in accordance with the relevant medical research rules of China in the study. Written informed consent regarding knowledge of adverse reactions to Gadolinium contrast was obtained from all patients prior to MR examination. All patient sensitive information was treated with strict secrecy and used solely for the purposes of this study.

### Study sample

**Figure 4:**
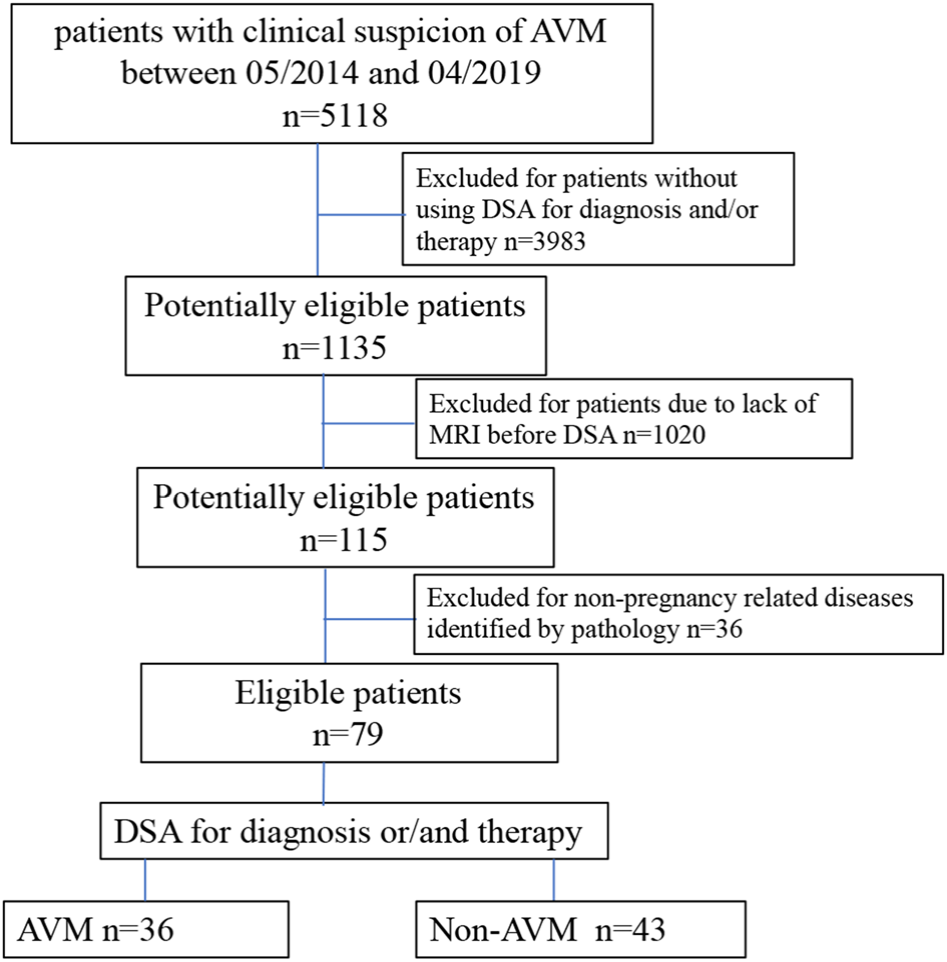
Flowchart showing the inclusion and exclusion criteria.

From May 2014 to April 2019, a total of 5118 consecutive women with clinical and ultrasound findings indicating a suspicion of pregnancy-related diseases (RPOC [309/5118], CSP [3800/5118], and GTD [1009/5118]) were reviewed from our institutional database. A total of 3983 patients were excluded because they had unavailable DSA data for diagnosis and/or therapy, were treated by transcervical hysteroscopic resection, D&C, an intravenous injection of methotrexate, or conservative treatment, or were not treated in our hospital. In addition, 1020 patients were excluded because MRI was not performed before DSA. Thirty-six patients were excluded because they had non-pregnancy-related diseases identified by pathology (haematoma or blood clot [12/36], myoma degeneration [5/36], infections [19/36]). Thus, 79 patients were finally included in the data analysis according to the following inclusion criteria (Fig. 4): (i) available MRI data acquired before DSA; (ii) available DSA data for diagnosis and/or therapy; and (iii) pregnancy-related diseases such as RPOC, GTD and CSP confirmed by pathology.

### Imaging protocol

MRI examination was performed for patients with RPOC, CSP, and GTD before they were managed with D&C, transcervical hysteroscopic resection or laparoscopic resection. All MRI examinations were performed using 1.5 T MRI systems (Philips Achieva 1.5 T with Nova Dual HP, Best, the Netherlands) using an 8-channel phased-array body coil. The MRI protocol included axial T1-weighted fast spin echo imaging, coronal and sagittal T2-weighted fast spin echo imaging, axial T2-weighted fast spin echo imaging with fat suppression and diffusion-weighted imaging (DWI). Contrast-enhanced MRI was performed for 22 patients using T1-weighted imaging (T1WI) with fat saturation was performed in the axial, sagittal and coronal planes after the intravenous administration of 0.2 ml/kg body weight gadopentetate dimeglumine (Gd-DTPA, Magnevist; Bayer Schering, Berlin, Germany). The scanning parameters were summarized in Table 4.

**Table 4:** Imaging parameters of sequences used in pregnancy-related diseases.

	Repetition time/echo time(ms)	Thickness (mm)	Intergap (mm)	Turbo factor	Field of view	Matrix	NEX
T1-weighted fast spin echo	575–460/10–15	5	1	3–5	320 × 320	246 × 246–375 × 375	4
T2-weighted fast spin echo	3000–3400/75–100	4	1	18–20	260 × 260–320 × 320	260 × 260–355 × 355	2 or 4
T2-weighted fast spin echo imaging with fat suppression	4000/100	4	1	18–20	260 × 260–320 × 320	260 × 260–355 × 355	2 or 4
Contrast-enhanced T1-weighted fast spin echo	497/10	5	1	3–5	260 × 260–300 × 300	320 × 320	2 or 4
Diffusion-weighted imaging (b-values, 0 and 800)	3000/75	5	1	–	260 × 260–320 × 320	260 × 260	2 or 4

Angiography and uterine artery embolization (UAE) were performed for 79 patients in our study using a therapeutic angiographic unit equipped with a digital flat panel detector system (Philips FD20 DSA) and nonionic contrast medium (iopamidol injection, 370 mg iodine per mL, Bracco Sine). All procedures were performed under conscious sedation (using midazolam and fentanyl) and local anaesthesia after a preprocedural evaluation performed by an anaesthesiologist. Moderate sedation (phenobarbital, 100 mg) and local anaesthesia were achieved with an intramuscular injection, and a 5-F vascular sheath (Radifocus; Terumo, Tokyo, Japan) was inserted into the right femoral artery using the Seldinger technique. Then, the uterine arteries were identified with DSA, and catheterization was performed selectively with a 5-F catheter (Radifocus; Terumo, Tokyo, Japan) along with an injection of 9 mL contrast material at a flow rate of 3 mL/s. Angiograms were obtained through the catheter, and special attention was paid to the draining or feeding branches. The material used for embolization in this study was either a gelatine sponge (560–710 µm, Alicon) or polyvinyl alcohol (500–710 microns, 1CC, COOK Incorporated), or both. The embolization was complete when the maximal reduction in flow or stasis was visualized within the system. A final aortogram was obtained to assess the size of any residual vascular malformations with an injection of 6–12 mL contrast material at a flow rate of 1 mL/s.

### Image analysis

For MRI, in our study, the imaging sequences included T1WI, T2WI, T2WI-fat suppression, DWI and contrast-enhanced series. The flow void sign was difficult to observe on the T2WI-fat suppression, DWI and contrast-enhanced series due to hypointensity in the parametrium as well as the flow void. Therefore, we mainly analysed the flow-void sign on T1 and T2 turbo spin echo (TSE) images without fat suppression or contrast medium. If any artifacts within the scan area that affected the display of flow-void sign on T1 and/or T2 TSE images, it should be excluded.

As previously described^15,28,32,35,36^, the flow-void sign was defined as multiple dotlike, tubular structures or multiple serpentine with low signal intensity located within the myometrium and parametrium on both T1WI and T2WI in our study. If the image quality is low and the flow-void sign is too small to observe, we could consider no flow-void sign within the myometrium and/or parametrium. In some cases, the dilated uterine artery may also show flow voids, but we do not need to discriminate the uterine artery from pathological vessels in the myometrium and/or parametrium.

On one hand, two experienced gynaecological radiologists (reader 1, with 8 years; and reader 2, with 12 years) who were not aware of the DSA results reviewed all MRI images in consensus to evaluate the following traits for each pregnancy-related disease: (a) the morphological characteristics of the lesion (including location, signal of MRI and the invasion of myometrium); (b) flow voids sign present vs. absent in parametrium and myometrium, respectively.

**Figure 5:**
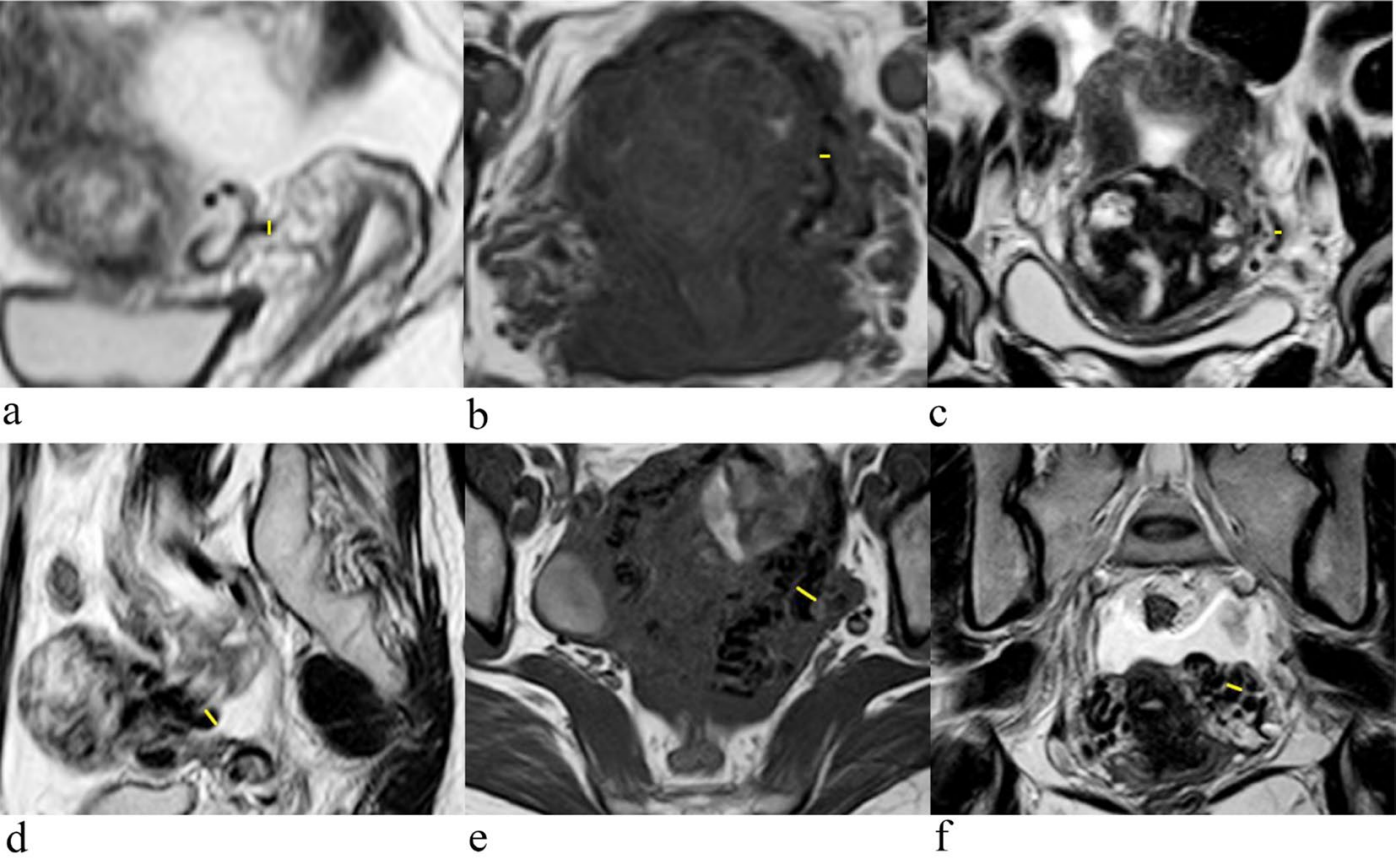
Images obtained of a 30-year-old woman with RPOC without uterine AVMs. The fv-D measurements in the parametrium on MRI. (**a**) Sagittal T2WI (fv-D = 2.3 mm). (**b**) Axial T1WI (fv-D = 2.0 mm). (**c**) Coronal T2WI (fv-D = 2.7 mm). The mean value is 2.3 mm. Images obtained of a 32-year-old woman with RPOC coexisting with a uterine AVM. The fv-D measurements in the parametrium on MRI. (**d**) Sagittal T2WI (fv-D = 5.5 mm). (**e**) Axial T1WI (fv-D = 5.4 mm). (**f**) Coronal T2WI (fv-D = 5.6 mm). The mean value is 5.5 mm.

On the other hand, reader 1 and reader 2 recorded the short diameter of the most prominent flow void (hereinafter referred to as fv-D) independently. The method of fv-D measurement is as follows: we chose the plane in which flow voids were the most prominent in the parametrium, then fv-D was measured in the coronal, sagittal and axial planes and then averaged and recorded. The same method was applied for measuring fv-D in the myometrium. If there was no flow void sign in the myometrium or/and parametrium, then we recorded the fv-D value as 0 mm. The measurements obtained for fv-D are shown in Fig. 5.

To assess interobserver reliability, the fv-D measurements were performed in a blinded fashion by reader 1 and reader 2. To evaluate intraobserver reliability, the reader 1 completed the first analysis of all images and then repeated the measurements a week later.

On DSA, as a reference standard, the presence of uterine AVMs was confirmed if there were one or more draining veins on early arterial-phase images of the angiography^21,37^. The blood supply arteries and drainage veins as well as the relationship between pelvic blood vessels were evaluated.

### Statistical analysis

Statistical analyses were performed with IBM SPSS Statistics version 25.0 and medcalc 19.11. Descriptive statistics included the mean and standard deviation (SD) for normally distributed variables. Categorial variables are expressed as numbers and probabilities. Fisher’s exact probability was used to compare the probability of flow void signs between patients with uterine AVMs and those without uterine AVMs. Additionally, the independent sample t-test was used to compare fv-D values in different locations between patients with and without uterine AVMs. Receiver operating characteristic (ROC) curves were plotted for fv-D in the myometrium and parametrium. The area under the curve (AUC), sensitivity, specificity, positive predictive value, negative predictive value, Youden index and cut-off value were calculated to determine which diagnostic indicator was superior. The cut-off value was determined based on the Youden index. Bland–Altman analysis was used to further assess intra- and interobserver agreement by calculating the bias (mean difference) and the 95% limits of agreement (1.96 standard deviations from the difference). Intraclass correlation coefficients (ICCs) were calculated to assess intraobserver and interobserver reliability using single measures for absolute agreement in a two-way mixed model. A two-tailed P value < 0.05 was considered statistically significant.
